# Targeting JNK-interacting protein 1 (JIP1) sensitises osteosarcoma to doxorubicin

**DOI:** 10.18632/oncotarget.600

**Published:** 2012-09-28

**Authors:** Jantine Posthuma De Boer, Pim W. van Egmond, Marco N. Helder, Renée X. de Menezes, Anne-Marie Cleton-Jansen, Jeroen A.M. Beliën, Henk M. W. Verheul, Barend J. van Royen, Gert-Jan J.L. Kaspers, Victor W. van Beusechem

**Affiliations:** ^1^ Department of Orthopaedic Surgery, VU University Medical Center, Amsterdam, the Netherlands; ^2^ Department of Surgery, Medisch Centrum Alkmaar (MCA), the Netherlands; ^3^ Research Institute MOVE/Skeletal Tissue Engineering Group Amsterdam (STEGA), the Netherlands; ^4^ Department of Epidemiology and Biostatistics, VU University Medical Center, Amsterdam, the Netherlands; ^5^ Department of Pathology, Leiden University Medical Center (LUMC), the Netherlands; ^6^ Department of Pathology, VU University Medical Center, Amsterdam, the Netherlands; ^7^ RNA Interference Functional Oncogenomics Laboratory (RIFOL), Department of Medical Oncology, VU University Medical Center, Amsterdam, the Netherlands; ^8^ Paediatric Oncology/Haematology, VU University Medical Center, Amsterdam, the Netherlands

**Keywords:** osteosarcoma, functional genomics, siRNA, chemosensitisation, JIP1

## Abstract

Osteosarcoma (OS) is the most common primary malignant bone tumour in children and adolescents. Despite aggressive therapy, survival outcomes remain unsatisfactory, especially for patients with metastatic disease or patients with a poor chemotherapy response. Chemoresistance contributes to treatment failure. To increase the efficacy of conventional chemotherapy, essential survival pathways should be targeted concomitantly. Here, we performed a loss-of-function siRNA screen of the human kinome in SaOS-2 cells to identify critical survival kinases after doxorubicin treatment. Gene silencing of JNK-interacting-protein-1 (JIP1) elicited the most potent sensitisation to doxorubicin. This candidate was further explored as potential target for chemosensitisation in OS. A panel of OS cell lines and human primary osteoblasts was examined for sensitisation to doxorubicin using small molecule JIP1-inhibitor BI-78D3. JIP1 expression and JIP1-inhibitor effects on JNK-signalling were investigated by Western blot analysis. JIP1 expression in human OS tumours was assessed by immunohistochemistry on tissue micro arrays. BI-78D3 blocked JNK-signalling and sensitised three out of four tested OS cell lines, but not healthy osteoblasts, to treatment with doxorubicin. Combination treatment increased the induction of apoptosis. JIP1 was found to be expressed in two-thirds of human primary OS tissue samples. Patients with JIP1 positive tumours showed a trend to inferior overall survival. Collectively, JIP1 appears a clinically relevant novel target in OS to enhance the efficacy of doxorubicin treatment by means of RNA interference or pharmacological inhibition.

## INTRODUCTION

Osteosarcoma (OS) is the most common primary malignant bone tumour in children and adolescents. The gold standard for therapy consists of a combination of multi-agent neoadjuvant chemotherapy, followed by radical surgery and adjuvant chemotherapy. With this aggressive regimen, 5-year survival rates of approximately 65% are obtained in patients with localised disease. However, in the case of metastatic or recurrent disease, 5-year survival rates are reduced to only 20% [1-4]. Chemoresistance, both intrinsic and acquired, is a key issue in the failure of current treatment to cure patients with OS [5,6]. A variety of combination therapy regimens and dose escalation of several therapeutics have not improved survival outcomes. Also, the current chemotherapy regimens are demanding for the patients and serious adverse effects, such as severe mucositis, bone-marrow depression and cardiotoxicity are often encountered. [7-10] In order to improve treatment efficacy whilst limiting adverse effects, new treatment strategies for OS are warranted.

Targeting essential survival pathways in combination with conventional therapy could be a strategy to improve the efficacy of current drug regimens. The identification of key regulators of drug response is an essential step in the design of such new targeted treatment strategies. RNA interference (RNAi)-based screening is a powerful technology for the discovery of candidate drug targets in malignant cells [11-13]. Proteins of the human kinome are involved in many cellular processes, including inter- and intra-cellular signalling, gene transcription, metabolism, cell shape and motility, proliferation, differentiation, survival and apoptosis. Kinases are known to play essential roles in disease development [14,15] and the kinome is therefore likely to harbour potential drug targets. Furthermore, kinases have been the subject of the design and development of small molecule drugs that target specific pathways in malignant cells [16-20]. This has confirmed the feasibility of targeting kinases with specific small molecules and their utility as targets for therapy. Here, we performed an siRNA screen of the human kinome to systematically identify genes that are involved in the survival of OS cells treated with doxorubicin. One candidate, MAPK8IP1, which encodes mitogen-activated protein kinase 8 interacting protein 1, also known as JNK-interacting protein 1 (JIP1), was further analysed for its potential use as a target for sensitisation of OS to doxorubicin.

## RESULTS

### siRNA screening identifies regulators of doxorubicin response in OS cells

**Figure 1:**
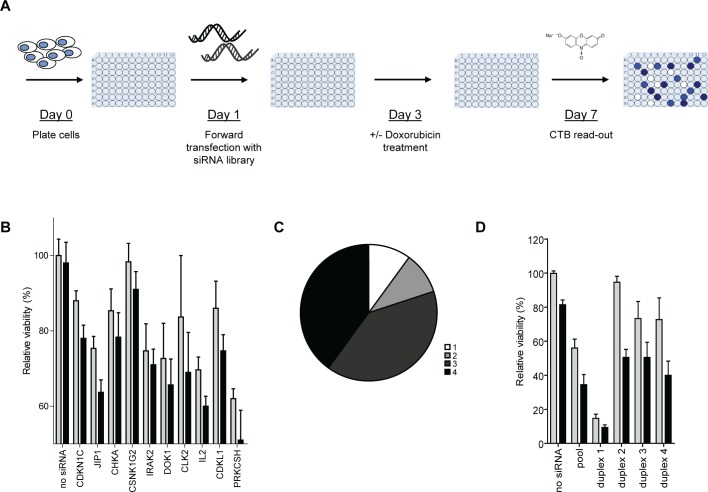
siRNA library screen of the human kinome identifies enhancers of doxorubicin response in OS (A) Schematic overview of the set-up of the screens performed with pools of 4 siRNAs against 788 human kinases and kinase-associated genes in SaOS-2 cells. (B) Screen results of the 10 selected candidate hits showing the effects of gene-silencing only (grey bars) versus gene-silencing + doxorubicin treatment (black bars) on cell viability. Bars represent the average cell viability measured in the 3 screens; error bars indicate standard deviations (SD). (C) Pie chart summarizing the secondary screen results. The chart shows the number of separate siRNA duplexes of 4 individual siRNAs tested per gene, that reproduced the doxorubicin-sensitising phenotype of the pooled siRNAs per gene. (Bar graphs for the separate siRNA duplexes are provided in panel D for JIP1 and *Supplementary Figure S1* for the other genes). (D) Confirmation of the doxorubicin-sensitising phenotype with siRNAs against JIP1. Cells were transfected with the indicated siRNA duplex and cultured in the presence (black bars) or absence (grey bars) of doxorubicin at IC20 concentration. Bars represent results from an experiment performed in triplicate; error bars indicate SD..

In order to identify regulators of doxorubicin response in OS, we performed cell viability screens on SaOS-2 cells using an siRNA library targeting the human kinome (Figure 1A). Screens were performed three times, each time including pairs of plates with and without doxorubicin treatment at an approximate IC20 concentration. Assay metrics based on mock- versus siPLK1-treated wells revealed Z'-factors ranging from 0.69 to 0.82 in the three experiments, indicating strong assay resolution [21]. *Supplementary Table S1* lists robust z-scores of all tested genes per screen. The effects of doxorubicin plus siRNA treatment were analysed using an empirical-Bayes linear model. *Supplementary Table S2* lists the computed treatment effects for all tested genes. Table 1 lists the genes that showed a most significant combination treatment effect (threshold p < 0.025). As indicator of the sensitising potential of gene silencing to doxorubicin treatment we calculated relative cytotoxicities (i.e., doxorubicin plus siRNA effect/doxorubicin effect). We then selected 10 candidate genes that met the following criteria: p < 0.025 and FDR < 0.4 and/or p < 0.025 and relative cytotoxicity > 3-fold. The mean relative cell viabilities of SaOS-2 cells treated with the selected siRNAs in the presence or absence of doxorubicin are shown in Figure 1B. siRNA against JIP1 appeared to elicit the most potent and highly significant enhancement of doxorubicin-induced cell kill (relative cytotoxicity = 8.6; p = 1.0*E-04; FDR = 2%). To confirm the findings in the primary screen for the 10 candidate genes, the candidates were reanalysed using 4 siRNAs directed against different sequences on their mRNA. For 8 candidate genes, the doxorubicin-sensitising phenotype could be reproduced with at least 3 individual siRNAs, suggesting that they represent genuine therapeutic targets (Figure 1C). Figure 1D shows the reanalysis results for JIP1. Three siRNAs (i.e., duplexes #2, #3, and #4) clearly enhanced cell kill after doxorubicin treatment, confirming the phenotype that was observed with the siRNA pool. In fact, these siRNAs exhibited a more selective effect, as they caused less direct cytotoxicity than the pool in the absence of doxorubicin. This could be explained by a profound cytotoxicity induced upon transfection of siRNA duplex #1. For this reason, siRNA duplex #1 was considered to elicit an off-target effect and was excluded from further analyses. Supplementary Figure S1 shows the reanalysis results of the remaining 9 candidate genes. For 7 candidate genes, i.e. CDKN1C, JIP1, CHKA, CSNK1G2, IRAK2, DOK1, CLK2 and IL2, the doxorubicin-sensitising phenotype could be reproduced with at least 3 individual siRNAs. Two genes could not be confirmed; CDKL1 had only 2 effective duplexes and PRKCSH was excluded from further analysis because three of the tested duplexes induced an increase in cell viability, yielding only 1 effective duplex for this gene.

**Table 1:** Primary screen hit list

Accession number	Gene symbol	Gene name	p-value	t-statistic	FDR	Relative cytotoxicity
NM_000076	CDKN1C	cyclin-dependent kinase inhibitor 1C (p57, Kip2)	6.3E-05	−4.00	0.02	5.4
NM_005456	JIP1 (MAPK8IP1)	mitogen-activated protein kinase 8 interacting protein 1	1.0E-04	−3.89	0.02	8.6
NM_212469	CHKA	choline kinase alpha	1.1E-03	−3.26	0.12	5.7
NM_001319	CSNK1G2	casein kinase 1, gamma 2	3.7E-03	−2.90	0.22	2.7
NM_001570	IRAK2	interleukin-1 receptor-associated kinase-like 2	3.8E-03	−2.89	0.22	8.0
NM_001381	DOK1	docking protein 1, 62kDa (downstream of tyrosine kinase 1)	5.3E-03	−2.79	0.28	4.4
NM_001291	CLK2	CDC-like kinase 2	7.1E-03	−2.69	0.35	1.9
NM_020421	ADCK1	aarF domain containing kinase 1	9.1E-03	−2.61	0.41	1.3
NM_031284	ADP-GK	ADP-dependent glucokinase	0.011	−2.55	0.44	2.0
NM_004196	CDKL1	cyclin-dependent kinase-like 1 (CDC2-related kinase)	0.011	−2.54	0.44	3.6
NM_000586	IL2	interleukin 2	0.013	−2.48	0.49	5.2
NM_014826	CDC42BPA(a)	CDC42 binding protein kinase alpha (DMPK-like)	0.016	−2.42	0.49	11.1
NM_052947	HAK (ALPK2)	alpha-kinase 2	0.016	−2.42	0.49	1.1
NM_198452	PNCK	pregnancy up-regulated non-ubiquitously expressed CaM kinase	0.020	−2.33	0.54	3.0
NM_001025778	VRK3	vaccinia related kinase 3	0.021	−2.31	0.55	1.9
NM_001001329	PRKCSH	protein kinase C substrate 80K-H	0.024	−2.26	0.61	7.0

The top 16 genes that, upon silencing elicit a statistically significant (p < 0.025) increase in sensitivity to doxorubicin treatment in osteosarcoma cells with corresponding p-values and t-statistics for treatment effect, and false discovery rates for each gene. Relative cytotoxicity is defined as doxorubicin plus siRNA effect/doxorubicin effect.

a excluded from further analysis due to high direct cytotoxicity upon siRNA treatment alone

### Depleting OS cells from JIP1 protein increases doxorubicin-induced cell death

**Figure 2:**
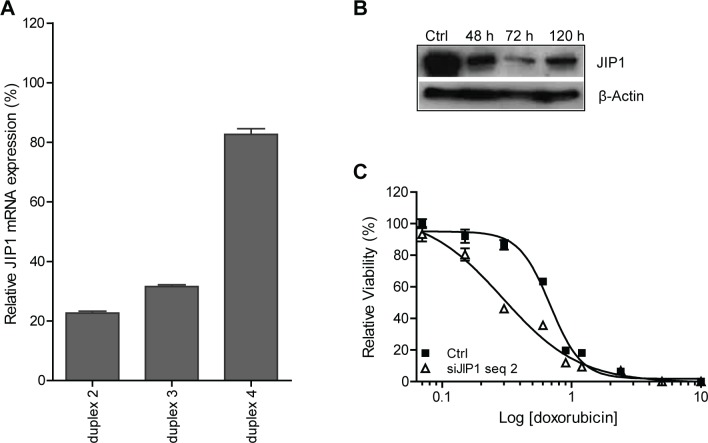
siRNA targeting JIP1 reduces JIP1 mRNA and protein expression in SaOS2 cells and causes increased sensitivity to doxorubicin (A) JIP1 mRNA silencing after transfection of JIP1 siRNA duplexes, analysed by RT-qPCR. JIP1 mRNA expression levels were normalised to GAPDH mRNA levels for each sample. Bars represent experiments performed in triplicate, error bars indicate SD. Results could not be obtained with JIP1 siRNA duplex #1 due to major cytotoxicity upon transfection of this siRNA. (B) Western blot analysis of JIP1 protein depletion at the indicated time-points after transfection of JIP1 siRNA duplex #2. (C) Dose-response curve of cells treated with doxorubicin (closed squares) and cells treated with doxorubicin after JIP1 gene silencing using JIP1 siRNA duplex #2 (open triangles). Results were obtained in an experiment performed in triplicate; error bars indicate SD. The JIP1 silenced cells show a significantly increased sensitivity to doxorubicin treatment (student's t-test at IC50; p < 0.0001).

Based on the siRNA screens, we selected JIP1 for further investigations. First, we assessed the gene-silencing efficiency obtained with the three functional JIP1 siRNA duplexes using qRT-PCR. Figure 2A shows that JIP1 siRNA duplex #2 was the most effective in suppressing JIP1 mRNA. Silencing JIP1 also depleted SaOS-2 cells of the protein product, as shown by Western blot analysis (Fig 2B). Decreased JIP1 protein expression became evident 2 days after transfection and was most pronounced at day 3. Doxorubicin dose-response curves for SaOS-2 cells and SaOS-2 cells transfected with JIP1 siRNA #2 (Fig 2C) demonstrated a significant shift in IC50, from 0.7 μg/mL for the control treated cells to 0.3 μg/mL for the JIP1 silenced cells (student's t-test, p < 0.0001). Hence, efficient siRNA-mediated silencing of JIP1 in SaOS-2 cells reduced JIP1 protein expression, sensitising the cells to doxorubicin treatment.

### Selective sensitisation of OS cells to doxorubicin-induced apoptosis using a small molecule JIP1 inhibitor drug

**Figure 3:**
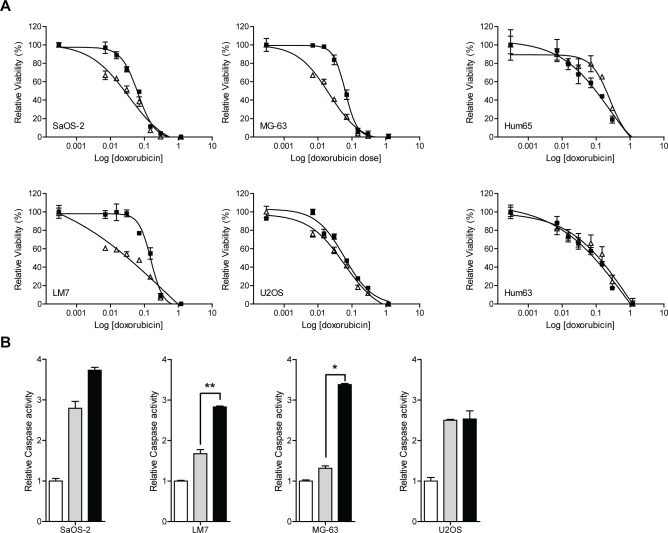
JIP1 inhibition sensitises OS cells but not normal osteoblasts to doxorubicin-induced apoptosis (A) Cells were treated in triplicate with doxorubicin (closed squares) or with doxorubicin and JIP1-inhibitor BI-78D3 (open triangles) and cell viability was determined four days later. Sigmoidal dose-response curves were created and IC50 values were calculated. The sensitising effect of JIP1 inhibition to doxorubicin treatment is significant in three out of four OS cell lines, (student's t-test at IC50; U2OS, p = 0.19, n.s; SaOS-2, p < 0.05; LM7 and MG-63, p < 0.0001). Human primary osteoblasts Hum63 and Hum65 do not show sensitisation to doxorubicin treatment in the presence of the JIP1-inhibitor (student's t-test at IC50; p = 0.459 and p = 0.417 respectively). (B) Caspase activation in OS cells treated with doxorubicin (grey bars) or with doxorubicin and JIP1-inhibitor BI-78D3 (black bars). White bars represent the control condition, which was set to 1. Bars represent an experiment performed in triplicate, error bars indicate SD. After combination treatment, caspase activity is distinctly higher in SaOS2, LM7 and MG-63 compared to doxorubicin treatment alone (student's t-test; LM7: **, p < 0.01; MG-63: *, p < 0.05; SaOS2, p = 0.08). In U2OS cells, caspase activity is comparable with and without JIP1 inhibition (p = 0.48).

Next, we investigated the effect of JIP1 inhibition using a small molecule drug on sensitivity to doxorubicin in OS cells and primary (non-malignant) human osteoblasts. To this end, cells were subjected to a dose range of doxorubicin concentrations in the presence or absence of a non-toxic dose (10 nM) of the small molecule JIP1-inhibitor BI-78D3 (22) that binds competitively at the JNK-binding site of JIP1. BI-78D3 increased sensitivity to doxorubicin treatment in 3 out of 4 tested OS cell lines, but not in primary osteoblasts (Fig 3A). IC50s were significantly decreased in SaOS-2 (p < 0.05), LM7 (p < 0.0001) and MG-63 (p < 0.0001) cells. To investigate if the combined effect of doxorubicin treatment and JIP1 inhibition was associated with stimulation of apoptotic cell death, we measured caspase-3 and caspase-7 activity in OS cells treated with doxorubicin in the presence or absence of 10nM BI-78D3. In all four OS cell lines, doxorubicin increased caspase activity (by 1.3 to 2.8-fold). In SaOS2, LM7 and MG-63 cells, caspase activity was further increased (to 2.5 to 3.8-fold) by addition of the JIP1-inhibitor (Fig 3B). This was significant in LM7 (p < 0.01) and MG-63 (p = 0.01) cells and approached significance in SaOS2 cells (p = 0.08). Contrarily, BI-78D3 did not augment caspase activation in U2OS cells (p = 0.48). Thus, the results of the caspase activity assay were in agreement with the observations made in the dose-response experiments. We therefore conclude that sensitisation of OS cells to doxorubicin treatment by JIP1 inhibition is probably caused by promotion of doxorubicin-induced apoptosis. Collectively, these data independently validate JIP1 as a regulator of doxorubicin response in OS and suggest that pharmacological inhibition of JIP1 could provide increased efficacy of doxorubicin treatment in OS, while sparing healthy bone cells.

### BI-78D3 inhibits JIP1-JNK interaction and JNK-phosphorylation in OS cells

**Figure 4:**
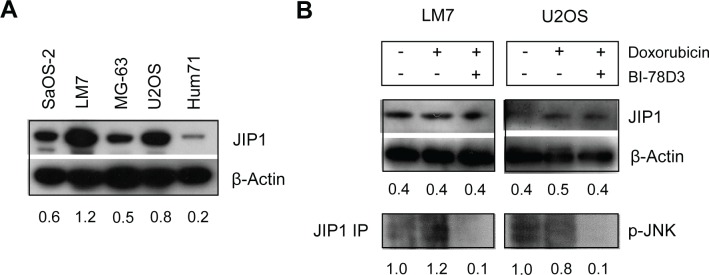
JIP1-inhibitor BI-78D3 reduces JIP1/p-JNK complexes in doxorubicin treated OS cells (A) Western blot analysis of baseline JIP1 levels in human OS cell lines and human primary osteoblast culture Hum71. All OS cell lines exhibit higher JIP1 levels than the human primary osteoblasts. (B) Immunoprecipitation and Western blot analysis of the JIP1/JNK activation module. LM7 and U2OS cells were treated with doxorubicin either in the presence or absence of BI-78D3. Whole cell lysates were analysed for JIP1 expression with β-actin serving as control for equal loading. In addition, cell lysates were immunoprecipitated with an anti-JIP1 antibody and Western blot analysis was done with an anti-p-JNK antibody. Doxorubicin-treated LM7 cells show an increased expression level of p-JNK compared to untreated cells, whereas in U2OS there is a slight decrease in p-JNK after doxorubicin treatment. After concurrent treatment with BI-78D3, p-JNK expression levels are diminished, indicative of inhibition of JIP1 scaffold function.

JIP1 is a scaffold protein that selectively mediates JNK signalling by assembling specific components of the MAPK cascade, including MLK, MKK4 and MKK7, in a signalling complex and JIP1 appears essential for JNK activation or maintenance of JNK phosphorylation. (22-28). In response to environmental stress, JNK is activated by phosphorylation of residues in its activation loop by MKK4 and MKK7, which in turn are activated by MLK. Apart from this, JIP1 has also been implicated in Akt1 activation and suppression of Notch1 activity. (29, 30). Western blot analysis demonstrated that JIP1 is expressed in all tested OS cell lines and at a lower level in primary osteoblasts (Fig 4A). We then investigated JIP1 expression and JNK phosphorylation in response to doxorubicin and BI-78D3 treatment in LM7 and U2OS cells. These cell lines respectively showed a strong and absent sensitisation to doxorubicin efficacy by treatment with JIP1-inhibitor BI-78D3. Treatment with doxorubicin did not affect JIP1 expression (Fig 4B). p-JNK could not be detected in whole cell lysates (not shown). However, upon immunoprecipitation with an anti-JIP1 antibody, p-JNK could be detected in both cell lines (Fig 4B). Doxorubicin treatment increased p-JNK in complex with JIP1 in LM7 cells, but not in U2OS cells. Concurrent treatment with BI-78D3 diminished p-JNK in both cell lines to undetectable levels, indicating successful inhibition of the JIP1-JNK interaction and JNK phosphorylation (Fig 4B). Together, these results suggest that inhibition of JIP1 increases the cytotoxicity of doxorubicin in cells that respond to doxorubicin treatment by increased JNK-signalling via assembly of the JIP1-JNK signalling complex.

### JIP1 is expressed in a majority of OS tumour specimens and is associated with poor survival

**Figure 5:**
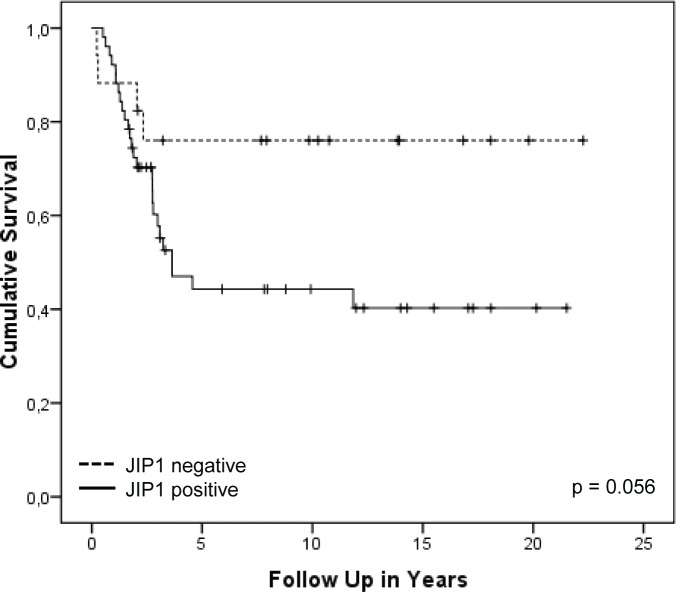
Kaplan-Meier overall survival analysis of OS patients with JIP1 positive or negative tumours Kaplan-Meier survival plot showing the cumulative survival of patients suffering from localised OS. Patients were divided into groups of patients with JIP1 positive tumours (55 samples) and those with JIP1 negative tumours (16 samples). The observed difference in cumulative survival between the two groups shows a trend towards inferior survival outcomes for patients with positive JIP1 staining (p = 0.056).

To assess the clinical relevance of JIP1 and to investigate if JIP1 could be considered a biomarker for OS, tissue micro arrays (TMAs) containing 647 cores of human primary OS samples (corresponding to 130 OS patients) were stained and analysed for JIP1 expression. Every tumour was represented by three cores on the TMAs and certain patients were represented more than once on the TMA i.e., with cores belonging to primary biopsies, first resections and metastases. Staining results were then correlated to clinical data. All samples were independently scored by two of the authors, with high observer agreement (*Supplementary Table S3*). Due to loss of tissue from the slide as a result of the cutting and staining procedures, 200 cores were unsuited for scoring, leaving 447 scored tissue cores. Of these, 67% were valued “positive” and 33% were valued “negative”. To study the predictive value of JIP1 staining on overall survival, we selected first biopsy or resection samples only; first biopsies had not been exposed to pre-operative chemotherapy and resection samples had been exposed to pre-operative chemotherapy. Samples of recurrences (both metastatic and local) were excluded to avoid confounding for inferior survival outcome as a result of metastatic and/or recurrent disease. After applying these criteria, data of 71 patients remained suitable for analysis. Figure 5 shows a Kaplan-Meier univariate analysis of JIP1 staining in tumour tissue as predictor of overall survival. Patients with JIP1 negative tumours showed a better survival outcome (mean 12.0 years) compared to patients with JIP1 positive tumours (mean 9.1 years); this difference in overall survival is borderline significant (LogRank, p = 0.056). JIP1 staining did not significantly correlate with event-free survival (p = 0.3) or with relapse (p = 0.2). Moreover, we did not find a significant association between JIP1 staining and response to multi-agent chemotherapy (p=0.9) (*Supplementary Table S4*). Thus, while JIP1 staining did not directly correlate to the response to chemotherapy, JIP1 positivity did show a strong trend towards inferior overall survival outcome, suggesting a possible role for outcome prediction in patients with OS. Importantly, JIP1 was found to be expressed in a majority of primary OS tumour samples, suggesting that JIP1 could be considered a clinically relevant target for treatment.

## DISCUSSION

In this work, we applied a functional genomics approach to systematically identify kinases and kinase-associated proteins that could be targets to enhance response to doxorubicin treatment in osteosarcoma. Doxorubicin is one of the major components of OS chemotherapy treatment, but its efficacy knows limitations in view of the 65% 5-year survival rate for patients with localised OS. Improving its efficacy could lead to enhanced tumour control or chemotherapy dose reduction in patients. The latter is relevant as doxorubicin is renowned for the serious adverse effects that regularly arise upon administration of this drug, especially cardiotoxicity (7). High throughput RNAi screens have previously been used successfully to identify essential oncogenes, modulators of response to anti-cancer drugs and cancer-specific targets for therapy (11-13, 31-33). Previous work in OS has led to the identification of potential therapeutic targets in OS by RNAi kinome library screening (34, 35).

In our siRNA library screen on SaOS-2 human OS cells, silencing of JIP1 led to the most potent and highly significant sensitisation to doxorubicin. This finding could be validated on SaOS-2 cells and two other p53-deficient OS cell lines using small molecule JIP1-inhibitor BI-78D3. OS cells expressed JIP1 at higher levels than normal osteoblasts and the sensitising effect of JIP1 inhibition to doxorubicin treatment was not observed in normal osteoblasts. This suggests that JIP1 is important for the survival of malignant, but not healthy cells subjected to chemotherapy, which makes JIP1 a potential target for selective anticancer therapy.

JIP1 is a scaffold protein that, in response to cellular stress, assembles a JNK activation module containing various kinases upstream of JNK, such as MKK4, MKK7 and MLK family members. The physical proximity between the JNK-signalling components in the JIP1/JNK signalling complex facilitates the phosphorylation of JNK and subsequent activation of the JNK signalling pathways. JIP1 inhibition is expected to alter or impair JNK-signalling, possibly leading to reduced c-Jun phosphorylation and downregulation of AP-1 transcriptional activation (22-27). JNK pathway activation is observed following cellular stress such as DNA damage, cytotoxic stress and γ-irradiation. However, the exact influence of JNK and AP-1 on cell death is ambiguous and reported to be cell and context dependent (6, 24, 27, 28, 36, 37). JNK has been found to promote as well as inhibit apoptosis, possibly depending on different upstream stimuli. In addition, JNK pathway activation has been suggested to promote p53-induced apoptosis. Hence, JNK might only exerts its anti-apoptotic effects in p53-deficient tumour cells (28). Our observations are in line with the described regulation of the JIP1/JNK signalling module. Treatment with doxorubicin induced JNK phosphorylation in p53 deficient LM7 cells, but not in p53 wild type U2OS cells, and inhibition of the JIP1-JNK interaction completely abrogated JNK phosphorylation. In addition, p53-null SaOS-2 and LM7 cells and p53-mutated MG-63 cells, but not p53 wild type U2OS cells (38-40) and normal osteoblasts, were sensitised to doxorubicin cytotoxicity upon JIP1 inhibition. Furthermore, increased cytotoxicity upon combination treatment was associated with promotion of apoptosis induction. This leads us to hypothesise that p53 deficient OS cells are sensitised to doxorubicin by inhibition of anti-apoptotic JNK signalling. However, given the relatively small number of cell lines analysed and the redundancy in survival pathways in tumour cells, this theory remains speculative.

The pharmacological verification of the chemosensitising effect of JIP1 inhibition in OS cells with BI-78D3 is promising in view of possible clinical translation of this treatment strategy, because whereas RNAi is a powerful, efficient method for the systematic discovery of drug targets, its applicability in a clinical setting is limited, mainly due to delivery issues. Although there is progression in the field of siRNA delivery techniques and some siRNAs are currently in clinical development, small molecule inhibitors generally have better pharmacokinetic properties and the road to clinical application seems less cumbersome than for siRNA (41). Additional treatment with a JIP1-inhibitor in OS patients receiving doxorubicin seems a realistic scenario that may elicit improved treatment efficacy and thus requires clinical studies.

To assess the potential clinical relevance of JIP1 expression in OS patients, we analysed human OS tissue samples by immunohistochemical staining of TMAs and correlated JIP1 staining to clinical outcome. At present, there is no specific predictive or prognostic marker in OS (42, 43). Prediction of treatment and/or survival outcomes might be of importance for the stratification of patients to treatment regimens and enable a more individualised treatment of OS patients by offering those patients additional targeted therapy when it could be anticipated that they will benefit from this specific treatment strategy. The importance of the discovery of reliable biomarkers in OS and their possible role in targeted treatment design has recently been highlighted by other research groups. (42-45). In this work, we were particularly interested in the correlations between JIP1 staining and survival and between JIP1 staining and chemotherapy response. Since, as demonstrated herein, silencing of JIP1 in OS cells enhances response to doxorubicin, high JIP1 expression levels in OS could perhaps correlate with inferior response to chemotherapy with doxorubicin and consequently inferior survival outcomes. In our tested dataset, we could not correlate JIP1 expression to inferior response to chemotherapy, possibly because all tested specimens were subdued to multi-agent chemotherapy and not doxorubicin mono-therapy. Also, given the presumed effect of JIP1 inhibition being dependent on defective p53, it might be worthwhile to analyse the tested specimens for p53 status. We did observe a distinct difference in overall survival between patients with positive and negative JIP1 staining, where positive JIP1 staining appeared associated with inferior survival outcome. While this finding needs confirmation in an independent dataset, our observation suggests that JIP1 could be a predictor for overall survival in patients with localised OS. Furthermore, JIP1 was found to be expressed in the majority of patient tissue samples, indicating that JIP1 is a relevant molecule in OS and that exploitation of JIP1 inhibition as additional treatment to current standard chemotherapy regimens could be beneficial to a majority of OS patients. In conclusion, based on our results, we propose JIP1 as a potential new drug target for OS to enhance the efficacy of chemotherapies including doxorubicin.

## MATERIALS AND METHODS

### Cell culture and compounds

Human osteosarcoma cell lines SaOS-2, MG-63, U2OS and LM7 (39) were kindly provided by Dr. F. van Valen (Westfalische Wilhelms-Universität, Münster, Germany), Dr. C. Löwik (Leiden University Medical Center, Leiden, the Netherlands), Dr. S. Lens (Netherlands Cancer Institute, Amsterdam, the Netherlands) and Prof. Dr. E.S. Kleinerman (MD Anderson Cancer Center, Houston, TX, USA), respectively. Human primary (short-term culture) osteoblasts Hum63, Hum65 and Hum71 were obtained from otherwise healthy patients undergoing total knee replacement after informed consent. All cells, with the exception of LM7, were cultured in D-MEM (PAA Laboratories) supplemented with 10% fetal calf serum (FCS) and 1 mg/mL Penicillin-Streptomycin (Gibco) at 37°C and 5% CO_2_ in a humidified incubator. LM7 was cultured in E-MEM (Lonza) supplemented with 10% FCS, 1 mg/mL Pen-Strep, 1% non-essential amino acids, 1% sodium pyruvate, 2 nM L-glutamine and 2% MEM-Vitamin solution (all: Gibco, Invitrogen) at 37°C and 5% CO_2_ in a humidified incubator. Doxorubicin (TEVA/Pharmachemie, Haarlem, the Netherlands) was diluted in D-MEM to the desired concentrations prior to use. The small molecule JIP1-inhibitor BI-78D3 (Sigma) (22) was dissolved in DMSO and diluted to the appropriate concentration in PBS directly prior to use.

### siRNA library screening

The siRNA screen was performed at the RNA Interference Functional Oncogenomics Laboratory (RIFOL) core facility of the VUmc Cancer Center Amsterdam, using an automated platform and the Human Protein Kinases ON-TARGET*plus* siRNA library from Thermo Fisher Scientific Dharmacon (Lafayette, CO). This library comprises siRNAs targeting 788 kinase and kinase-associated genes. Four different siRNA duplexes designed against each target gene were pooled and arrayed for screening in 96-well plate format. siRNA against PLK1 was used as a positive control for cell death and mock treated wells served as negative controls. For confirmation studies, individual siGENOME siRNAs (TFS Dharmacon) were used. Forward transfections were done according to the manufacturer's recommendations, using 25 nM siRNA and 0.05% (v/v) Dharmafect 1 transfection reagent (TFS Dharmacon) in 100 μL culture medium. Figure 1A shows a schematic overview of the screen design. The screens were performed three times, each time including one set of plates with doxorubicin and one without. SaOS-2 cells were plated at a density of 2,000 cells/well in 96-well plates (Greiner) and transfected with siRNAs the next day. Two days post-transfection, doxorubicin was added to one set of plates to a final concentration of 30 ng/mL; culture medium was added to the other set. Four days after the start of doxorubicin treatment, cell viability was determined using the CellTiter-Blue (CTB) Cell Viability Assay (Promega), measuring fluorescence at 540 nm excitation and 590 nm emission wavelengths using a Tecan Infinite F200 Microplate Reader (Tecan Trading AG, Switzerland). The CTB Cell Viability Assay measures metabolic capacity of viable cells by reduction of the indicator dye resazurin into fluorescent resofurin through the action of cellular enzymes, in which the measured fluorescent signal is proportional to the number of viable cells (www.promega.com).

Plate data was read and configured in R (The R Project for Statistical Computing) (46) using the cellHTS2 software package (47). We used the negative control-medians per plate to center plate-specific log2 intensities and then computed robust z-scores per screen. The z-score matrix containing 3 untreated and 3 treated screens was used to study the combination treatment effect (i.e., combined effect of doxorubicin and siRNA as discriminated from single agent effects) by means of an empirical- Bayes linear model, using the limma software package (48). The obtained p-values for the combination treatment effect were then corrected for multiple testing using Benjamini & Hochberg's step-up false discovery rate (FDR) (49). The magnitude of sensitisation to doxorubicin by siRNA transfection was estimated by calculating the ratio of mean cytotoxicity observed after combination treatment over mean cytotoxicity observed after doxorubicin treatment.

### Doxorubicin dose response analysis and apoptosis assay

Human OS cells or primary osteoblasts were plated at a density of 2,000 cells/well in 96-well format and transfected with JIP1 siRNA as described above or incubated with BI-78D3 at a non-toxic dose of 10 nM. Two days after siRNA transfection or concurrently with BI-78D3 addition, cells were treated with a doxorubicin dose range and four days later cell viability was assessed using the CTB assay as described above. To analyse apoptosis, OS cells were plated in white opaque 96-wells plates and treated with doxorubicin (0.1 μg/mL) or combination treatment with doxorubicin plus BI-78D3 (10nM). At 24h post-treatment, caspase activity was measured using the Caspase-Glo 3/7 assay (Promega) according to the manufacturer's instructions. Luminescence read-out was performed using a Tecan Infinite F200 Microplate Reader (Tecan Trading AG, Switzerland). Results were analysed using GraphPad Prism^®^ Version 5.01 (GraphPad Software Inc.). Relative caspase activity was normalised to the signal measured in the control (PBS) condition.

### Quantitative RT-PCR

Cells were plated, allowed to adhere and transfected with siRNA as described above. Two days post-transfection cells were harvested by trypsinisation. Cellular RNA was isolated using the RNeasy Kit (Qiagen) and quantified by spectrophotometry using the NanoDrop ND-1000 and ND-1000 software version 3.3 (Isogen Lifescience). Per sample, 1 μg total RNA was reverse transcribed into cDNA using the SuperScript® III RT kit plus Random Primers (Invitrogen). Real time quantitative PCR for MAPK8IP1 / JIP1 was performed using the Quantitect primer assay designed for detection with SYBR-Green (Qiagen). Amplification was measured on a LightCycler® 480 and analysed using the corresponding software, Version 1.5 (Roche). Relative MAPK8IP1 / JIP1 gene expression was normalised to that of GAPDH using the ΔΔC_t_ method (50).

### Immunoprecipitation and Western blot analysis

Baseline JIP1 protein expression levels across OS cell lines and primary osteoblasts and JIP1 downregulation at the protein level 1-5 days after transfection of SaOS-2 cells was assessed using Western blot. Cells were lysed in buffer containing Protease and Phosphatase Inhibitor Cocktails (Sigma). Proteins were quantified with the BCA protein Assay Kit (Pierce). A total of 30 μg protein per sample was separated on a SDS-PAGE gel and transferred to a PVDF membrane. Blots were incubated with primary antibodies rabbit-anti-MAPK8IP1 (181-193) at a dilution of 1:1,000 (Sigma-Aldrich) and mouse-anti-β-actin (Abcam) at a dilution of 1:10,000, followed by secondary antibody incubation with HRP-conjugated goat-anti-rabbit and goat-anti-mouse immunoglobulins (DAKO), respectively. Protein detection and visualisation was performed using ECL^+^ Western Blotting Detection Reagents (Pierce).

Regulation of JIP1 and JNK phosphorylation following doxorubicin and JIP1-inhibitor BI-78D3 treatment was analysed by immunoprecipitation followed by Western blot analysis. OS cells and human primary osteoblasts were plated and treated with doxorubicin (0.3 μg/mL) or combination treatment with doxorubicin plus BI-78D3 (10 nM) for 4 h and then lysed in phospho-lysis buffer (HEPES containing 0.5% β-glucose, 0.1% DTT, 0.1% Na_3_VO_4_) containing Protease and Phosphatase Inhibitor Cocktails (Sigma). For immunoprecipitation, 25 μL of IgG-sepharose beads (Pierce) were incubated with 2 μL rabbit-anti-MAPK8IP1 antibody for 1 h at 4°C under continuous motion. The antibody-bead conjugates were collected by centrifugation, mixed with 60 μg protein lysate for 1 h at 4°C under continuous motion. Immunoprecipitates were collected by centrifugation. Sample separation and transfer followed as described above. Primary antibody incubation was done using rabbit-anti-phospho-JNK (Cell Signalling) and secondary antibody incubation and protein detection were performed as described above. Protein levels were quantified using the Image J tool (National Institute of Health, USA). Intensities were normalised to β-actin levels in the Western blots and to the internal control sample in the immunoprecipitation.

### Tissue micro arrays and immunohistochemistry

Two tissue micro arrays (TMAs) containing a total of 647 cores of human primary OS samples (corresponding to 130 OS patients) and 20 control tissue cores, were stained and analysed for JIP1 expression (*Supplementary Figure* S2A) and then correlated to clinical and survival data. The TMAs were crafted at the Leiden University Medical Center (LUMC, Leiden, the Netherlands) according to the protocol described in *Mohseny* et al. (51). All patients were treated for OS at the LUMC in the period between 1984-2009. Available clinical data includes: age, gender, location and side of the primary tumour, response to chemotherapy according to the Huvos grading system (52) (when available), metastasis, recurrence, date of recurrence, survival, date of death (when applicable) and time of follow-up. (*See Supplementary Table S4*) The tissue array slides were heated at 60°C for 20 minutes prior to deparaffinization in Xylene and rehydration in a graded series of alcohol. Endogenous peroxidase activity was inhibited by incubation with 0.3% H_2_O_2_ diluted in methanol for 30 minutes. The arrays were boiled in 10 mM citrate buffer (pH 6) for 10 minutes and subsequently rinsed in PBS. The slides were incubated with rabbit-anti-MAPK8IP1 (181-193) primary antibody at a dilution of 1:500 O/N at 4°C. Antigen visualisation was performed using the EnVision^+^ Poly-HRP IHC Kit (Immunologic) and DAB chromogen solution. Slides were counterstained with hematoxylin, dehydrated and mounted.

### Tissue micro array scoring

The stained TMA slides were automatically scanned using a digital whole slide scanning system (Mirax slide Scanner system 3DHISTECH Ltd., Budapest, Hungary), equipped with a numerical aperture of 0.75 and a Sony DFW-X710 Fire Wire 1/3” type progressive SCAN IT CCD (pixel size 4.65 × 4.65 μm), with an actual scan resolution (effective pixel size in the sample plane) at 20x objective of 0.23 μm. All 647 samples were independently examined and scored by two of the authors (JP and PE). The scoring was performed using dedicated TMA scoring software (3DHISTECH Ltd., Budapest, Hungary) in a blinded fashion. To facilitate the scoring and improve the reproducibility of scoring, a consensus chart with exemplary staining patterns per category was created (*Supplementary Figure S2B*) and used by the observers during the scoring of the samples. The staining per tissue was assessed and valued as “negative” or “positive”. Due to loss of cores during the cutting and staining procedure, not all cores could be included for analysis. Samples were considered unsuitable for scoring when less than 30% of tissue was present on the digital copy of the tissue core. In case of insufficient tissue, the cores were given the value “no data”. Of each tumour, three cores are present on the TMA. The grading scale consistend of 3 values (0 = no data; 1 = negative; 2 = positive) To assure a robust staining score, i.e. reliable scoring per tumour sample, we applied a threshold of a minimum of 6 scores 1 or 2 (excluding “no data” observations) for tumours to be included in the statistical analysis. We used the mean of the scores to assign the final staining result (positive or negative) to a sample. The clinical data and the staining results were entered and statistically analysed in SPSS, version 17.0 (SPSS Software, Inc., Chicago, IL, USA). To assess inter- and intra-observer agreement in grading JIP1 staining, Kappa statistics were used. Because inter- and intra-observer reproducibility may be biased by an overemphasis on patients with grade 0 findings, kappa values were therefore also calculated with the exclusion of grade 0 findings (censored Kappa). Values between 0 and 1 were interpreted according to modified published guidelines (53, 54) (*Supplementary Table S3*). Kaplan-Meier analysis was used to assess survival and differential survival between groups was analysed using the Log Rank test. To determine significant differences between categorical groups, the Pearson chi-square test was used. In numerical groups, the independent t-test and one-way ANOVA were used. The threshold for statistical significance was set at p < 0.05.

## Supplementary Figures and Tables




