# Supplementary material for: Targeting JNK-interacting protein 1 (JIP1) sensitises osteosarcoma to doxorubicin
Source: Oncotarget. 2012 Sep 28;3(10):1169–81. doi: 10.18632/oncotarget.600 (PMC3717953; doi:10.18632/oncotarget.600)
Supplement: Supplementary file 1 [file oncotarget-03-1169-s001.pdf]

Targeting JNK-interacting protein 1 (JIP1) sensitises osteosarcoma to doxorubicin - Posthuma De Boer et al

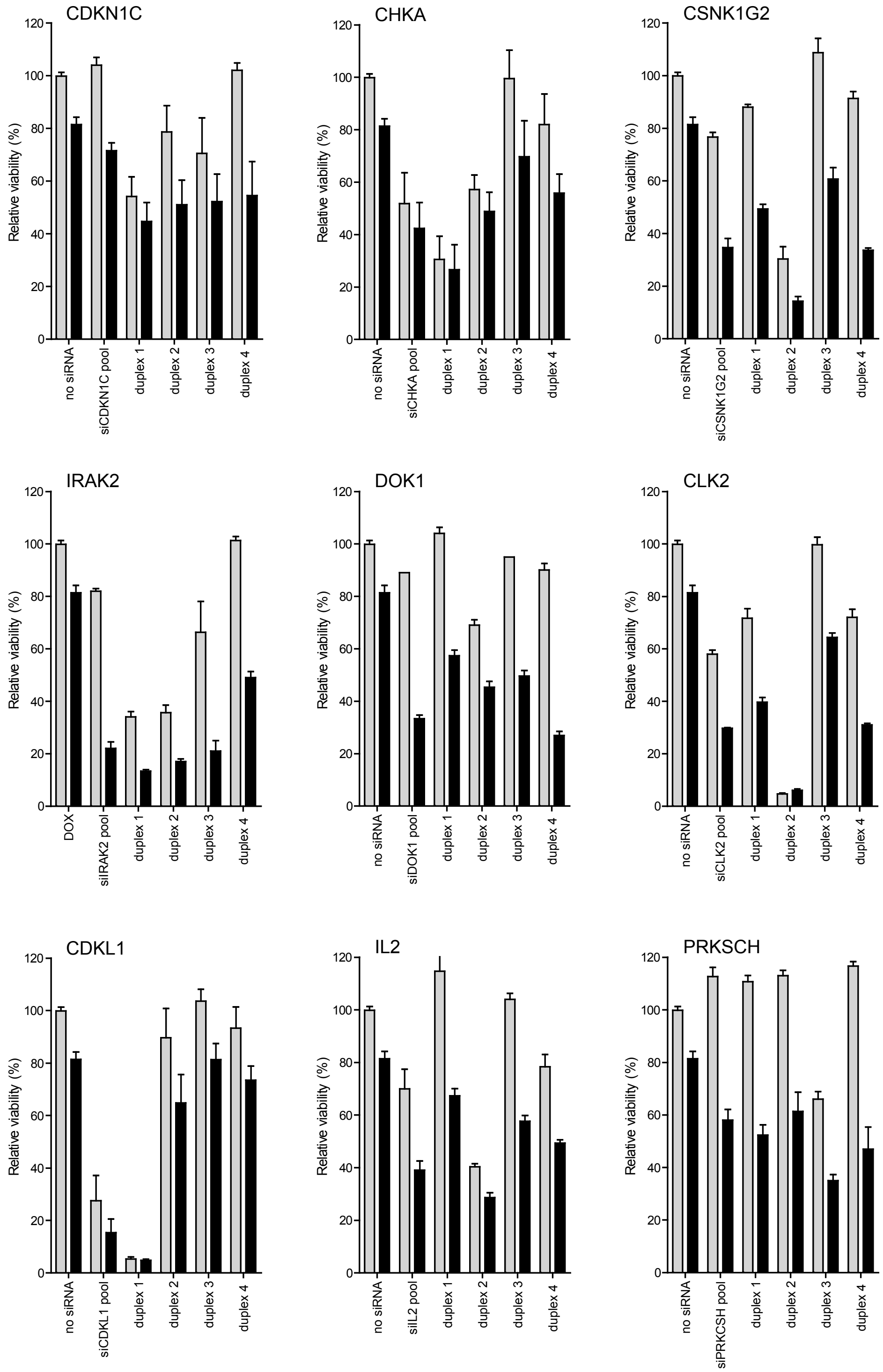

Supplementary Figure S1: siRNA deconvolution analysis of selected screen hits. Results for JIP1 are shown in Figure 2 of the main manuscript; the other nine selected hits are shown here. Confirmation scores of all 10 analyses are summarised in Figure 1B of the main manuscript. Two genes could not be confirmed: CDKL1 has 2 effective duplexes and PRKCSH was excluded from further analysis since gene-silencing alone induced an increase in cell viability in 3 duplexes, which was considered to be undesirable and leaving one effective duplex for this gene.

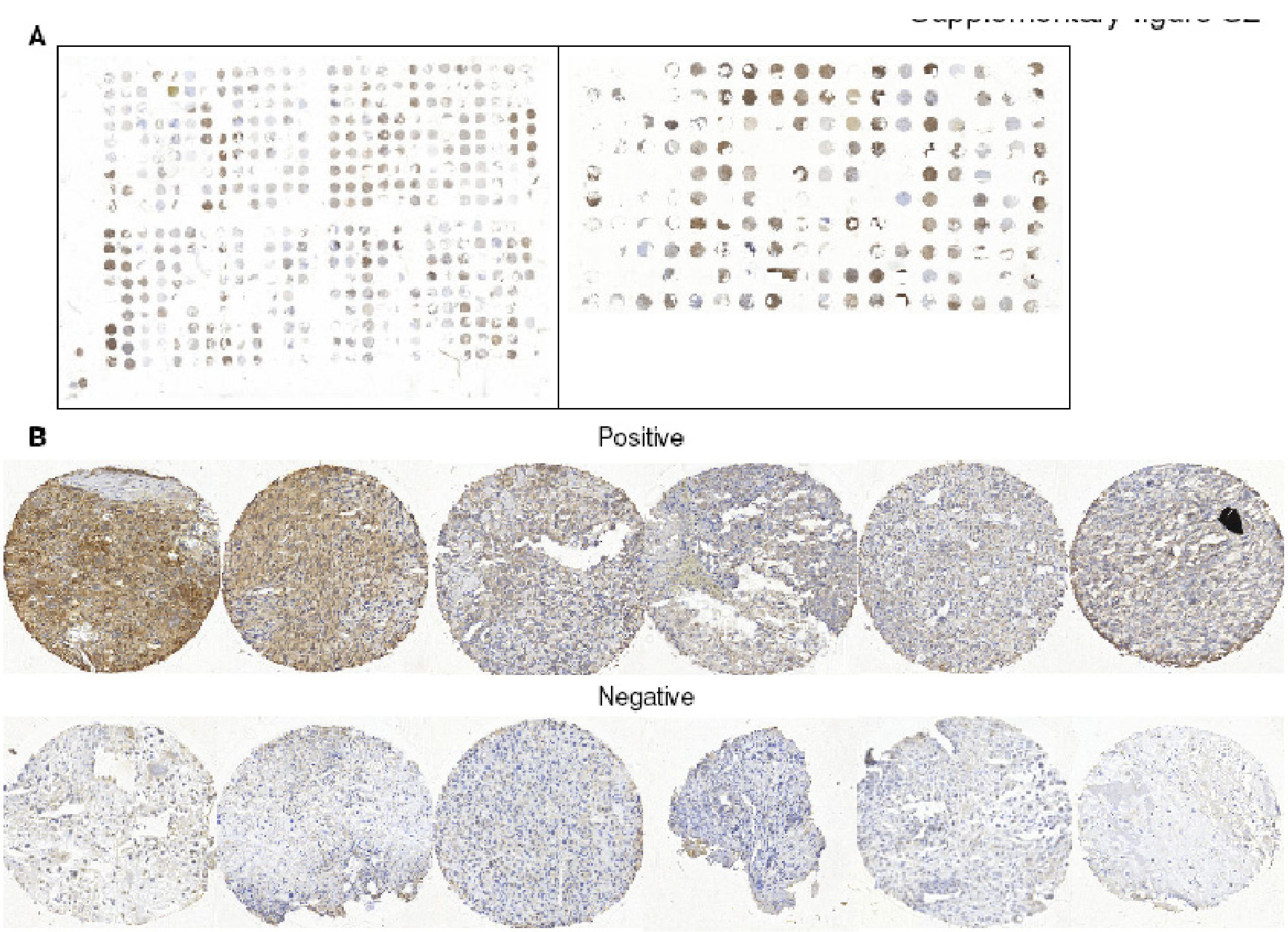

Supplementary Figure S2: Tissue micro array (TMA) layout and staining consensus chart. (A) Overview of the layouts of the two tissue micro arrays analysed for JIP1 staining. Corresponding clinical data is summarised in Supplementary Table S3. (B) Consensus chart showing six exemplary tissue cores per category (negative or positive) used as guide while scoring JIP1 staining.
